# Matrix Metalloproteinase 9 (MMP-9) Regulates Vein Wall Biomechanics in Murine Thrombus Resolution

**DOI:** 10.1371/journal.pone.0139145

**Published:** 2015-09-25

**Authors:** Khanh P. Nguyen, Kirk C. McGilvray, Christian M. Puttlitz, Subhradip Mukhopadhyay, Christine Chabasse, Rajabrata Sarkar

**Affiliations:** 1 Division of Vascular Surgery, Oregon Health and Science University, Portland, OR, United States of America; 2 Orthopaedic Bioengineering Research Laboratory, Department of Mechanical Engineering and School of Biomedical Engineering, Colorado State University, Fort Collins, CO, United States of America; 3 Center for Vascular and Inflammatory Diseases and Division of Vascular Surgery, University of Maryland, Baltimore, MD, United States of America; University of Louisville, UNITED STATES

## Abstract

**Objective:**

Deep venous thrombosis is a common vascular problem with long-term complications including post-thrombotic syndrome. Post-thrombotic syndrome consists of leg pain, swelling and ulceration that is related to incomplete or maladaptive resolution of the venous thrombus as well as loss of compliance of the vein wall. We examine the role of metalloproteinase-9 (MMP-9), a gene important in extracellular remodeling in other vascular diseases, in mediating thrombus resolution and biomechanical changes of the vein wall.

**Methods and Results:**

The effects of targeted deletion of MMP-9 were studied in an *in vivo* murine model of thrombus resolution using the FVB strain of mice. MMP-9 expression and activity significantly increased on day 3 after DVT. The lack of MMP-9 impaired thrombus resolution by 27% and this phenotype was rescued by the transplantation of wildtype bone marrow cells. Using novel biomechanical techniques, we demonstrated that the lack of MMP-9 significantly decreased thrombus-induced loss of vein wall compliance. Biomechanical analysis of the contribution of individual structural components showed that MMP-9 affected the elasticity of the extracellular matrix and collagen-elastin fibers. Biochemical and histological analyses correlated with these biomechanical effects as thrombi of mice lacking MMP-9 had significantly fewer macrophages and collagen as compared to those of wildtype mice.

**Conclusions:**

MMP-9 mediates thrombus-induced loss of vein wall compliance by increasing stiffness of the extracellular matrix and collagen-elastin fibers during thrombus resolution. MMP-9 also mediates macrophage and collagen content of the resolving thrombus and bone-marrow derived MMP-9 plays a role in resolution of thrombus mass. These disparate effects of MMP-9 on various aspects of thrombus illustrate the complexity of individual protease function on biomechanical and morphometric aspects of thrombus resolution.

## Introduction

The biology of venous thrombus resolution plays a central role in a number of significant cardiovascular diseases. Deep venous thrombosis (DVT) is an exceedingly common and serious problem with between 350,000 to 600,000 cases annually in the U.S. and 200,000 deaths from pulmonary embolism (PE) [1]. The acute mortality of PE can be effectively prevented by early diagnosis of DVT and prompt anticoagulation. However, despite anticoagulation, post-thrombotic syndrome develops in 20–60% of patients within months to years after initial DVT [1]. Post-thrombotic syndrome consists of pain, swelling and skin ulceration that impair daily activities, prevent employment and lower quality of life. Patients with more rapid spontaneous thrombus resolution of DVT have less subsequent venous reflux [2], suggesting that the process of thrombus resolution (or failure thereof) is central to the pathogenesis of post-thrombotic syndrome.

There is no specific therapy to prevent or treat post-thrombotic syndrome. Defining the cellular and molecular mechanisms of venous thrombus resolution is critical to developing such therapy to potentially restore vein wall structure and function after thrombosis. Experimental thrombus resolution is defined as decreasing thrombus mass [3] but there has been more emphasis recently on the importance of thrombus-induced inflammation, fibrosis and stiffening of the vein wall [4] [5]. Experimental and clinical studies have shown that thrombus resolution induces fibrosis and stiffening of the vein wall, decreasing vein wall compliance and capacitance of the vein [6] [7] [8]. Increasing vein wall stiffness (decreasing compliance) involves endogenous plasmin [9] and can be altered by heparin treatment [5]. Vein wall fibrosis studies are limited to immunohistochemical analyses in mice [10] [11]. Rigorous biomechanical analysis of the roles of individual genes in mediating changes in wall compliance are lacking.

Studies from our laboratory and others have shown that thrombus resolution induces expression and activity of matrix metalloproteinases (MMP’s), a family of proteases important in angiogenesis, tissue remodeling and cell migration [12] [13] [14]. MMP-9 regulates vascular remodeling including ischemia-induced angiogenesis, aortic aneurysm development and atherosclerotic plaque rupture [15] [16] [17]. MMP-9 expression and activity are increased during thrombus resolution *in-vivo* [12] [13]. MMP-9 plays a key role in macrophage migration [18] and macrophages are a critical cell type in thrombus resolution [19]. One prior study demonstrated that MMP-9 increased vein wall collagen and expression of other pro-fibrotic genes during thrombus resolution, however the functional (biomechanical) significance of these changes was not assessed [11]. We recently reported novel techniques to measure biomechanical properties of mouse vein wall that allows reproducible detection of the changes in vein wall induced by thrombus resolution [20]. The biomechanical effects of thrombus resolution have been studied mostly in a rat model [8], which can identify vein wall fibrosis and stiffening due to thrombus resolution but cannot define the role of individual genes. We hypothesized that macrophage-derived MMP-9 regulates macrophage migration and thrombus resolution and that MMP-9 is involved in the fibrosis and vein wall stiffening induced by thrombus resolution. By using biaxial micro-mechanical analysis of vein walls after thrombus resolution in mice lacking MMP-9, we are able to identify distinct effects of MMP-9 on thrombus-induced remodeling of the extracellular matrix and collagen fibers. MMP-9 has opposing effects on resolution of thrombus mass and thrombus-induced stiffening of the vein wall, and also affects macrophage infiltration and collagen deposition.

## Materials and Methods

### Mice

Homozygous null MMP-9^-/-^ mice [21] on FVB background strain with targeted disruption of the MMP-9 gene (B6.FVB(Cg)-MMP-9tm1Tvu/J, Jackson Laboratories, Sacramento, CA) were used at age 10 to 12 weeks and weighed approximately 20 g at time of operation. Experiments were done in strict accordance with the Guide for the Care and Use of Laboratory Animals of the NIH and approved by our Institutional Animal Care and Use Committee at the Department of Veterans Affairs Medical Center, San Francisco, California by the Animal Studies Subcommittee (Protocol Numbers 08-047-01, 08-049-01, and 06-055-02). We used MMP-9^-/-^ and MMP-9^+/+^ homozygous animals derived from matings of MMP-9^+/-^ heterozygotes to eliminate possible effects of genetic drift within the colony. These littermate controls were used in each group of experiments. Animal experiments were done under general anesthesia and all efforts were made to minimize suffering. All animals were housed under controlled conditions for temperature and humidity in a specific pathogen free (SPF) facility, using a 12 h light/dark cycle and had continuous access to food and water. The number of animals required for each experiment (N = 6–12) was estimated based on our previous experience and mice were randomly assigned to each treatment group.

### Surgical Model of Deep Vein Thrombosis

**Fig 1:**
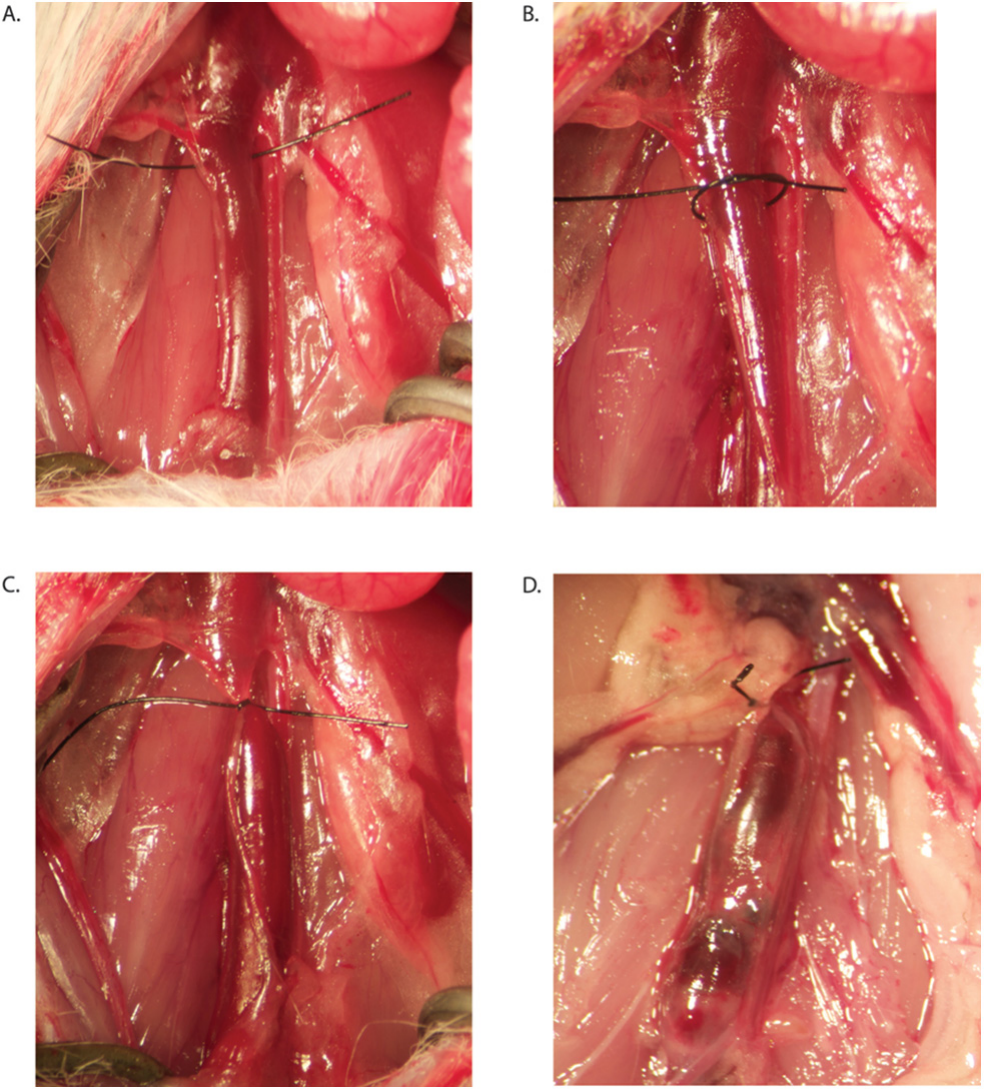
Mouse model of deep venous thrombosis. A-C. Ligation of the infrarenal inferior vena cava (IVC) is accomplished with a monofilament suture. D. Representative thrombus after ligation of IVC.

To study MMP-9 expression and function, we used the well-established stasis model of thrombus resolution in the mouse [22]. Animals were removed from their housing to the laboratory where all experiments were conducted during daylight hours. Analgesia and anesthesia doses are chosen as were recommended by our veterinarians and animal care facility staff. Under buprenorphine (0.05–0.1 mg/kg subcutaneous) and isofluorane gas anesthesia (1–5% inhalant to effect administered with precision vaporizer), the inferior vena cava (IVC) was ligated below the level of the renal veins and lumbar side branches cauterized as previously described (Fig 1) [12]. Control mice underwent sham surgery involving caval dissection and cautery of lumbar side branches to control for surgical inflammation and possible MMP activation.

To harvest tissue specimens, animals were sacrificed by isofluorane inhalation at an overdose followed by cervical dislocation to ensure death. Veins with thrombi were harvested at various time points after surgery on days 3, 8 and 12. Thrombus mass was normalized to body weight to control for differences in development and body mass between knockout strains. As previously defined, thrombus resolution was defined as the change in the mass of the thrombus and vena cava between day 3 and day 12, which in this model is approximately 50% [22]. Although the thrombus and vena cava are not separable after day 7, this 50% reduction is largely in the mass of the thrombus, as studies in sham-operated animals indicate minimal changes in the mass of the vena cava wall.

Additional specimens were harvested on days 2, 4, and 7 to analyze vein walls and thrombi separately to obtain expression and activity of MMP-9 over time. These time points were chosen because after 7 days it is not possible to separate the adherent thrombus to the vein wall due to the robust inflammatory response.

### Biaxial Stretch Experiments

For biomechanical analysis, thrombi were harvested on day 7 before the thrombus becomes surgically inseparable from vein walls secondary to inflammation [22] and prepared for testing as previous described [20]. Biaxial stretch experiments were designed to assess the role of MMP-9 expression on vein wall mechanics following IVC ligation and thrombus resolution.

Biaxial experimental data served as the input for strain energy function (SEF) modeling. The strain energy function model assumed material anisotropy, highly nonlinear stress-strain relationships and large deformations. Parameterized strain energy function coefficients served to demonstrate the relevant material changes secondary to DVT and to elicit the biomechanical effects of MMP-9 expression on vein wall mechanics following thrombus formation [20].

Creation of post-phlebetic veins involved complete ligation of the murine inferior vena cava (IVC), inducing stasis laminar thrombi as described above in MMP-9 wildtype and MMP-9 knockout mice. Thrombi were harvested after one week after surgery was chosen because thrombi become surgically inseparable from the vein wall after 7 days [22] secondary to intense inflammatory reaction.

Samples were prepared for biomechanical testing as previously described by McGilvray et al. [20], where the IVC was transected longitudinally, along the flow direction creating a planar sheet of vascular tissue. Briefly, a longitudinal venotomy was made in the IVC over the site of the thrombus and then the thrombus was separated from the vein wall. The vein was mounted to the biaxial device in a trampoline-like fashion using vascular suture (7–0 suture, 0.03mm diameter, monofilament, Surgipro II, Syneture). The stretch axes of the testing device where aligned with the axial or flow direction (AD) and circumferential direction (CD) of the specimen [23]. Previous work by our lab has demonstrated that these techniques can be used to measure localized tissue deformations and the resultant stress on the tissue with high fidelity and repeatability.

It was assumed that a “stress free” configuration could be approximated in a global sense through an ‘opened-up geometry’ [20, 24, 25, 26]. Samples were allowed to equilibrate under zero appreciable measured loads at the boundary for thirty minutes while completely immersed in saline to allow the sample to conform to a global stress-free and strain-free configuration [27]. The sample’s geometry was calculated from calibrated length measurements at five equally-spaced locations along the vessel (ImageJ 1.38x, National Institutes of Health, Bethesda, MD) [20].

Previously described biaxial stretch protocols were used to assess the biomechanical response of the tissue. These protocols were derived from a normal strain component-controlled test, where the ratio of the percent elongation were kept constant [20, 23, 28].

Each specimen was subjected to four biaxial protocols in which the ratio of the percent elongation along each axis, i.e. (λ_Axial Direction (AD)_-1: λ_Circumferential Direction (CD)_-1), was kept constant, where the change in stretch protocols were 1:1, 1:2, 0:1, and 1:0. To account for the inherent viscoelastic response of the tissue samples were cycled twenty times for each loading protocol, and data from the 20^th^ loading cycle was used for analysis and coefficient parameterization. Data collected during the 1:1 and 1:2 stretch protocols were used for parameterization of SEF material coefficients, and the data collected during the 0:1 and 1:0 stretch protocols were used to check the predictive capability of the material parameters derived from the constitutive model [20].

### Strain Energy Function Modeling

Strain energy function (SEF) modeling was performed to analyze the direct mechanical contributions of structural fibers of the vein wall. This SEF has a micromechanical basis, which takes into account the directions and dispersion of the primary fiber families and allows for direct comparisons between mechanically relevant vein wall constituents and the SEF coefficients. Five components (C_10_, K_1_, K_2_, κ, γ) contributing to vein wall stiffness were analyzed: extracellular matrix (C_10_), collagen and elastin fibers (K_1_), three-dimensional alignment of fibers (κ), angle of fiber orientation (γ), and fiber linearity (K_2_) as previously described [20].

For this analysis venous tissue was assumed to be a nonlinear, incompressible, anisotropic hyperelastic biomaterial [24]. The strain energy function (SEF) proposed by Gasser et al. [26] was selected. This SEF has a micromechanical basis, which takes into account the directions and dispersion of the primary fiber families. This is advantageous because it allows for direct comparisons between mechanically relevant vein wall constituents and the SEF coefficients [25, 26]. For completeness, the SEF can be expressed as:

$$\Psi ({Ī}_{1},{Ī}_{4},{Ī}_{6})=C_{10}({Ī}_{1}-3)+\frac{\kappa_{1}}{2\kappa_{2}}\overset{2}{\underset{\alpha =1}{\sum }}\left[e^{{\kappa_{2}[\kappa ({Ī}_{1}-3)+(1-3\kappa )({Ī}_{\beta }-1)]}^{2}}\right];\;\beta =4,6$$ Eq 1

where C_10_, K_1_, K_2_, and κ are material parameters (K_1_ has dimensions of stress; K_2_ and κ are dimensionless structural parameters). The expression of the SEF (ψ) (Eq 1) represents the two mechanically relevant constituents; the non-collagenous, isotropic ground substance or extracellular matrix, and the contributions from the different fiber families [25, 26]. For this analysis, where there are two families of fibers relevant to this analysis, therefore alpha (α) in Eq 1 ranges from 1 to 2.

The strain invariants are directly calculated from the primary stretch ratios as:

$${Ī}_{1}=\lambda_{axial}^{2}+\lambda_{circumferential}^{2}+{\left(\lambda_{axial}\lambda_{circumferential}\right)}^{-2}$$ Eq 2

$${Ī}_{4}={Ī}_{6}=\lambda_{axial}^{2}\sin^{2}\gamma +\lambda_{circumferential}^{2}\cos^{2}\gamma$$ Eq 3

where γ is a structural parameter denoting the angle of fiber orientation between the fiber families. Shear stretch contributions was neglected [20].

The principle Cauchy stresses (σ_α_, where α = 1 for the circumferential direction and, α = 2 for the axial direction) are derived from the SEF:

$$\sigma_{\alpha }=J^{-1}\lambda_{\alpha }\frac{\delta }{\delta \lambda_{\alpha }}\;\Psi ({Ī}_{1},{Ī}_{4},{Ī}_{6})=\lambda_{\alpha }\frac{\delta }{\delta \lambda_{\alpha }}\;\Psi ({Ī}_{1},{Ī}_{4},{Ī}_{6});\;\alpha =1,2$$ Eq 4

The measured stretch data from the 1:1 and 1:2 protocols and the corresponding Cauchy stress data for each sample were simultaneously fit to the above constitutive relation using a Levenberg-Marquardt nonlinear curve-fitting algorithm (MATLab, MathWorks, Natick, MA).

Fiber dispersion, the parameter kappa (κ) was parameterized in the range 0/18 ≤ κ ≤ 6/18 at 1/18 increments, and the fiber orientation parameter gamma (γ) was parameterized in the range 0 deg ≤ γ ≤ 45 deg at 15 degree increments [20]. The remaining parameters K_1_, K_2_, and C_10_ were determined from the curve fitting algorithm when the maximum sum of the squares residual value between the experimental and strain energy-based Cauchy stresses matrices was at a minimum over the range of kappa and gamma [20]. The parameters K_1_ and C_10_ have units of stress and give a measure of the biomechanical response of the fiber families (i.e., collagen and elastin) and the extracellular matrix, respectively. The parameters K_2_ is a unit less parameter that demonstrated the linear response of the fiber components in the vein wall. A statistically significant change in these parameters is an indicator that there has been a significant alteration in the biomechanical function of the related physiologic components.

### Western Blotting and Zymography

Thrombi and vein walls were snap frozen with liquid nitrogen and stored at -80 degrees Celsius. Samples were homogenized in lysis buffer on ice and protein content of lysates was quantified. For western blotting, equal amounts of protein were loaded per well and electrophoresed on 12% SDS–polyacrylamide gel and transferred to polyvinylidene difluoride membrane. The membrane was blocked and then probed with MMP-9 antibody (Santa Cruz Biotechnology, SC-6840, goat polyclonal IgG raised against a peptide mapping at the C-terminus of the MMP-9 of human origin, dilution 1:1000). Secondary antibodies were added, and bands were detected using an enhanced chemiluminescence detection kit. Western protein standards were used for molecular weight estimation on western blots.

For zymography, aliquots of lysates were loaded on 7.5% SDS-polyacrylamide gels containing gelatin and separated by electrophoresis with zymographic molecular weight standards.

Protein expression and gelatinolytic activity were quantified by densitometry using Image J software (U.S. National Institutes of Health, Bethesda, MD, USA, http://imagej.nih.gov/ij/).

### Morphometric Analysis

Tissues were fixed with 4% paraformaldehyde at 4 degrees Celsius, dehydrated in 70% ethanol, and embedded in paraffin for sectioning. Using standard immunohistochemical techniques, slides were stained for macrophages (Mac-3, BD Pharmingen, 550292, rat anti-mouse Mac-3 raised against mouse C57Bl/6 peritoneal exudate cells, dilution 1:500), endothelial cells (vWF, Chemicon International, AB7356, rabbit polyclonal antibody raised against human vWF purified from plasma, dilution 1:200 to 1:100), and collagen (picrosirius red, Fisher Scientific). For each sample, 6 high powered fields (HPF) were analyzed using ImageJ software (U.S. National Institutes of Health, Bethesda, MD, USA, http://imagej.nih.gov/ij/).

### Bone Marrow Transplantation Experiments

Bone marrow transplants were performed between the various MMP-9^+/+^ and MMP-9^-/-^ cohorts as indicated. Five week old congenic female recipient mice were irradiated with a single dose of 10 Grey and injected retro-orbitally with 25,000,000 donor cells from male donor mice. The bone marrow homing efficiency of transplanted donor cells was measured by polymerase chain reaction (PCR) analysis of recipient bone marrow from recipients for the presence of murine Y chromosome using the following specific primers: forward 5’TTGAGTTTTCTGTGTTTATCTTTGGCTGTC 3’ and reverse 5’TCTTCAGAGGTATTGTCTTCTACATGGCAG 3’. PCR products were separated on 1% agarose gel for optimal visualization. Four weeks after bone marrow transplantation, vena caval ligation was performed as described above and thrombus resolution analyzed.

### Statistics

Statistical analyses were computed using Prism (GraphPad Software Inc., La Jolla, CA) and SigmaStat 3.1 software (Jandel Scientific Software, San Jose, CA). Unpaired Student’s t-tests or one-way analysis of variance were used to compare groups that were normally distributed. Values are expressed as the mean ± standard deviation. The level of significance was set at p<0.05.

## Results

### MMP-9 expression and function are induced during thrombus resolution

**Fig 2:**
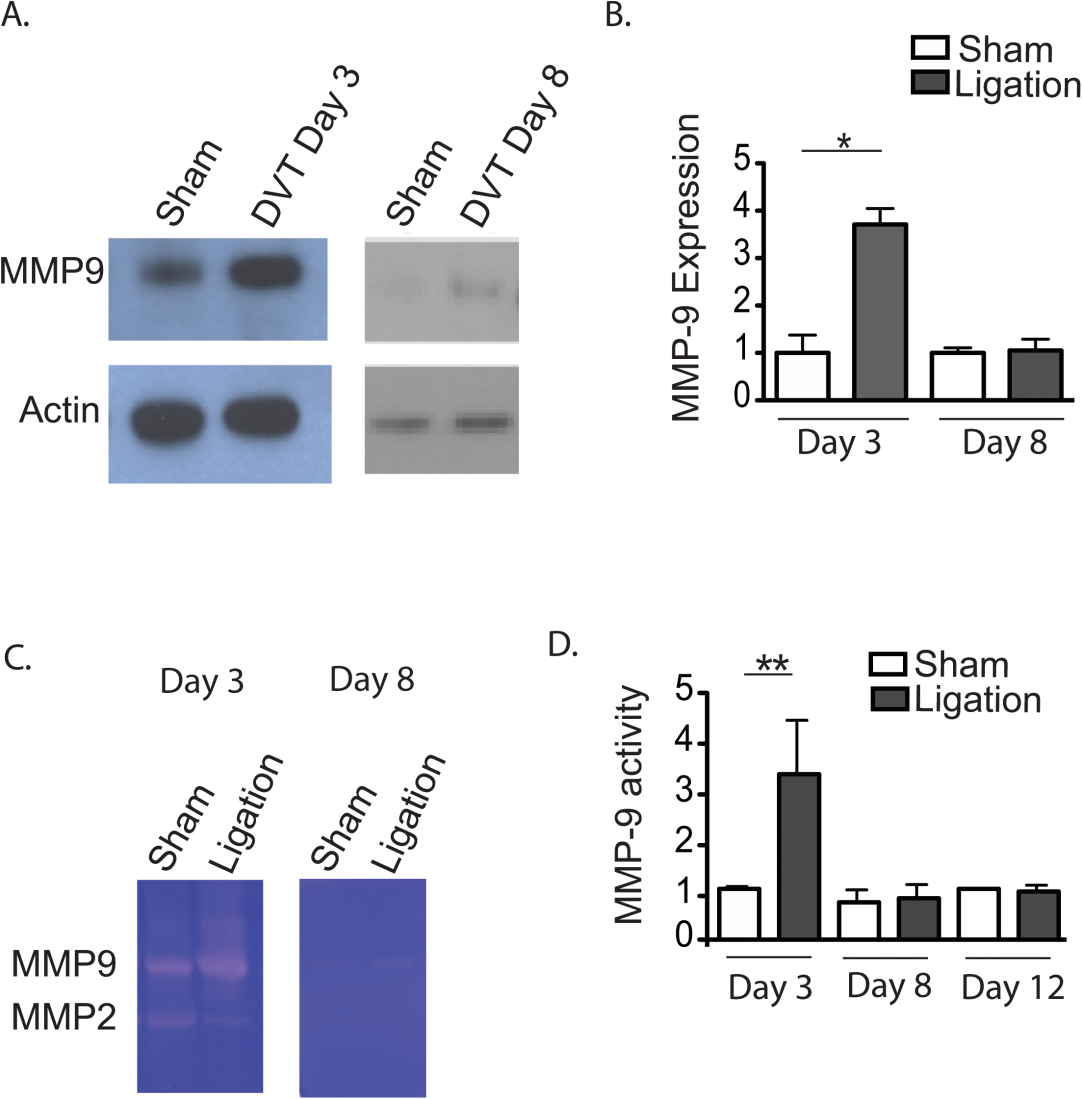
Expression and enzyme activity of MMP-9 are increased during thrombus resolution. MMP-9 expression by immunoblot analysis of combined thrombus and vein wall samples at day 3 and day 8 (A) and over time by densitometry analysis (B) in sham and ligation (DVT) groups. MMP-9 activity by zymography in combined thrombus and vein wall samples at day 3 and day 8 (C) and over time by densitometry analysis (D). Differential expression (E) and activity (F) of MMP-9 in vein wall (VW) versus thrombus (Th) at indicated time points. MMP-2 also indicated on zymogram (F). (*,**p<0.05)

**Fig 3:**
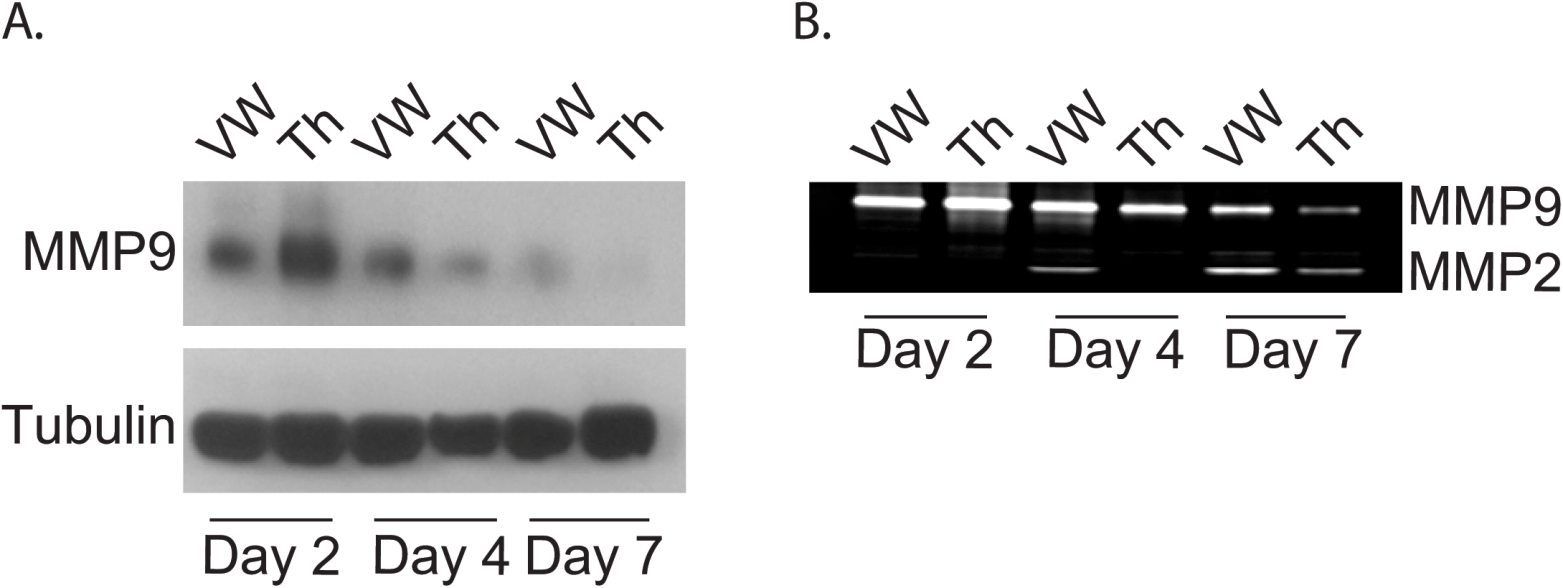
Expression and activity of MMP-9 in the vein walls versus thrombi. Differential expression (A) and activity (B) of MMP-9 in vein wall (VW) versus thrombus (Th) at indicated time points, **days 2, 4, and 7**. MMP-2 also indicated on zymogram (B) **on days 2, 4, and 7**. (*,**p<0.05).

We first studied the expression and activity of MMP-9 in the vein wall and thrombus during thrombus resolution (Fig 2). Compared with sham surgery, thrombus resolution induced MMP-9 expression and activity with a peak at day 2–3. MMP-9 expression was greater in the thrombus at day 2, but expression and activity were more sustained in the vein wall at later time points (day 7) when expression and activity in thrombus were undetectable. MMP-2 activity was induced later than MMP-9, and was stronger in the vein wall. (Fig 3)

**Fig 4:**
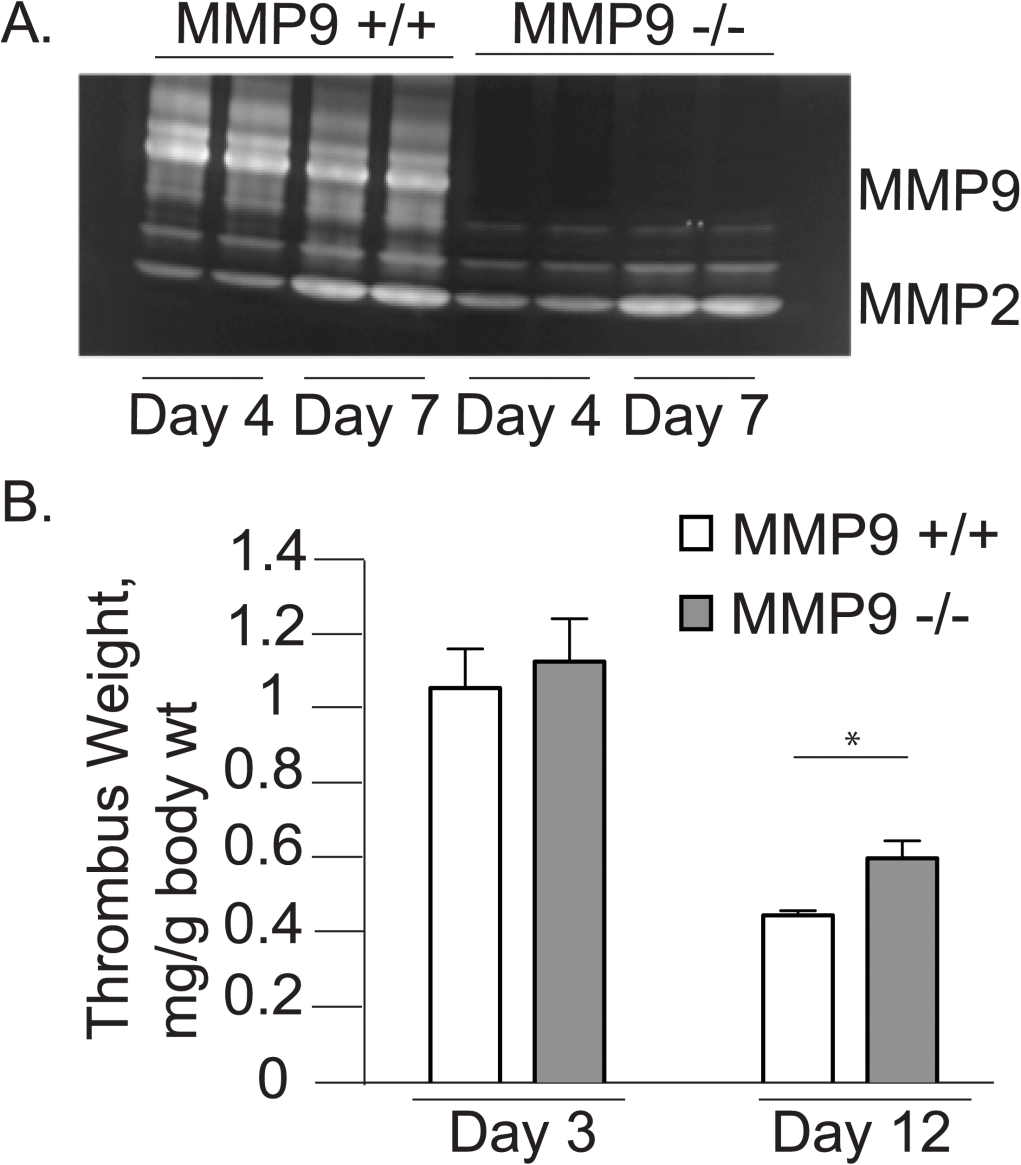
MMP-9 deletion results in impaired thrombus resolution. A. Representative zymograms showing MMP activity in combined thrombus and vein wall samples in MMP-9^+/+^ and MMP-9^-/-^ mice on day 4 and 7 after IVC ligation (DVT). B. Thrombus weights compared on days 3 (MMP9 +/+ 1.04±0.23 g/kg versus MMP9 -/- 1.11±0.26 g/kg; N = 5 per group, p = 0.67) and 12 (MMP9 +/+ 0.44±0.12 g/kg versus MMP9 -/- 0.56±0.1 g/kg, N = 6 per group; *p<0.05).

No MMP-9 activity was detectable in MMP-9^-/-^ animals (Fig 4A). There was a modest compensatory increase in MMP-2 activity in MMP-9^-/-^ mice which is consistent with previous reports [10]. MMP-9^-/-^ mice had significantly larger thrombus weights compared to control mice on day 12 demonstrating impaired thrombus resolution (MMP-9^+/+^ 0.010±0.001 g versus MMP-9^-/-^ 0.013±0.002 g, p<0.05). As transgenic MMP-9^-/-^ mice were significantly smaller than their age-matched wildtype controls (20.4±0.7 g versus 25.0±2.9 g, N = 6 per group, p<0.05), we also analyzed thrombus weight normalized to body weight and found that the effect of MMP-9 persisted when corrected for body mass (Fig 4B). Thrombus weights of MMP-9^+/+^ and MMP-9^-/-^ mice were not significantly different on day 3, demonstrating that the differences noted on day 12 were not due to differences in thrombus formation.

### Biomechanical Results: MMP-9 deletion improves post-thrombotic vein wall compliance

**Fig 5:**
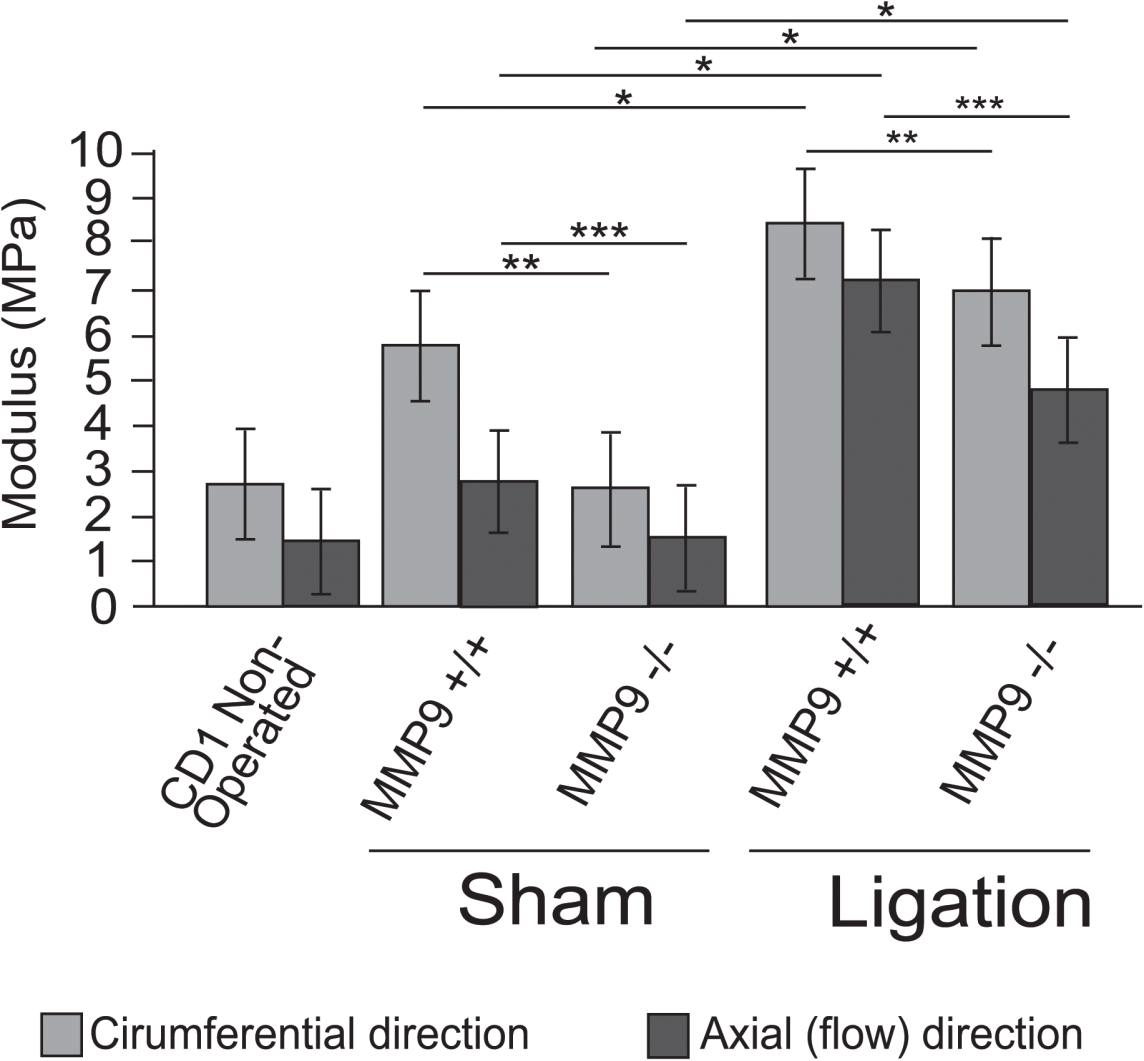
Biomechanical effects of MMP-9 deletion on the post-thrombotic vein wall. Overall biomechanical compliance (MPa) of vein walls after sham or ligation (DVT) in circumferential and axial directions from MMP-9^+/+^ and MMP-9^-/-^ mice (N = 8–12 per group; *,**,***p<0.05).

**Fig 6:**
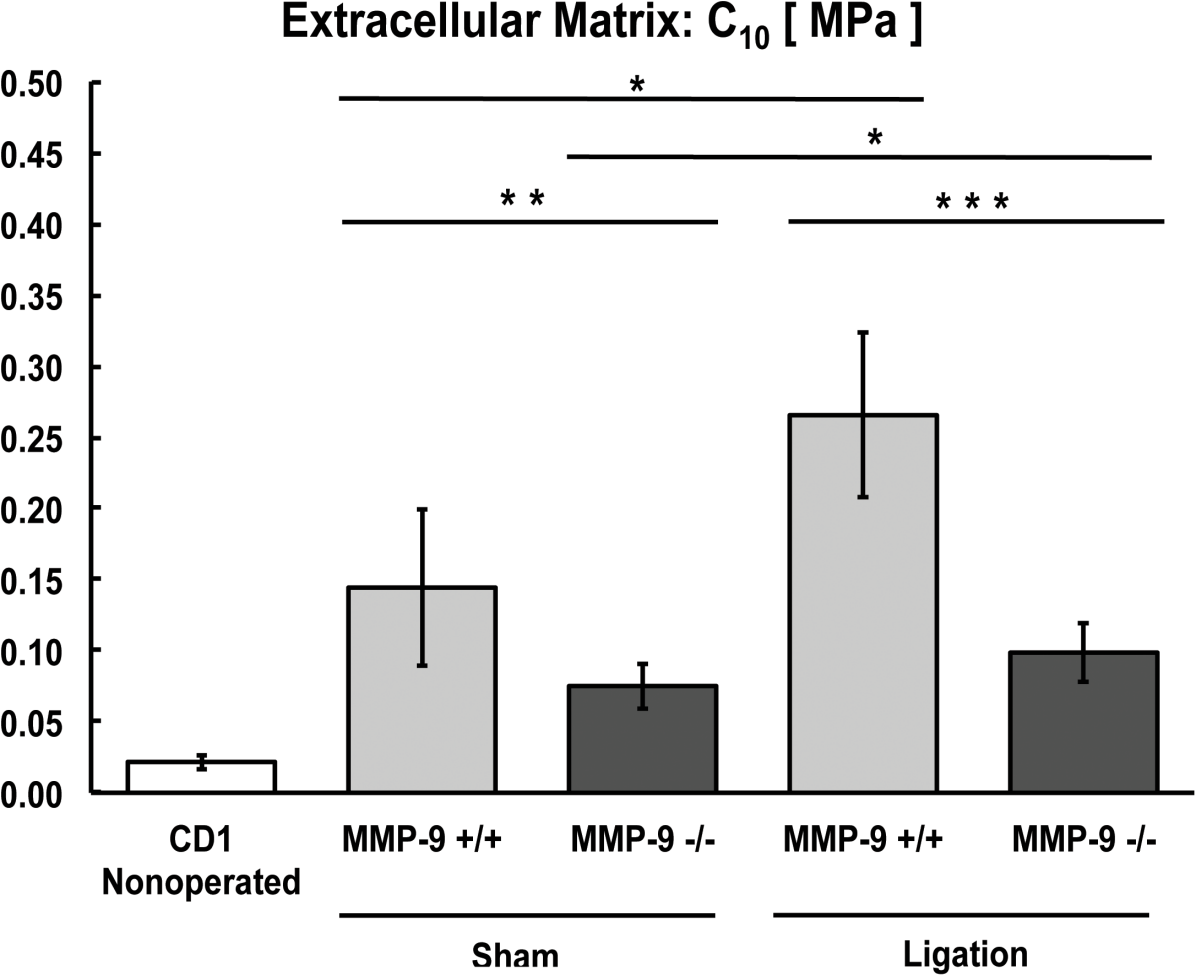
Biomechanical modeling of extracellular matrix (C_10_) from MMP-9^+/+^ and MMP-9^-/-^ mice (N = 8–12 per group; *,**,***p<0.05).

**Fig 7:**
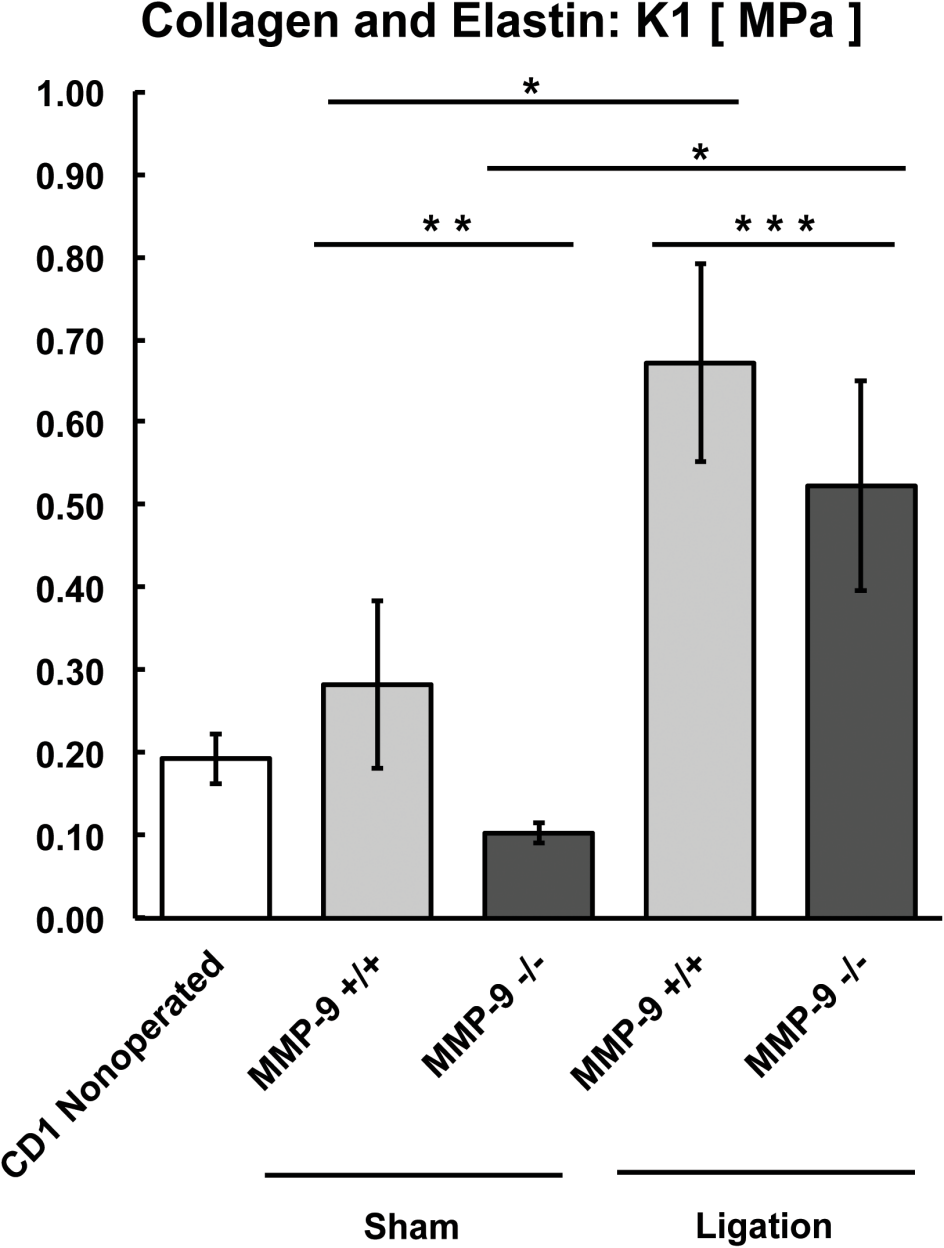
Biomechanical modeling of collagen and elastin (K1) from MMP-9^+/+^ and MMP-9^-/-^ mice (N = 8–12 per group; *,**,***p<0.05).

**Fig 8:**
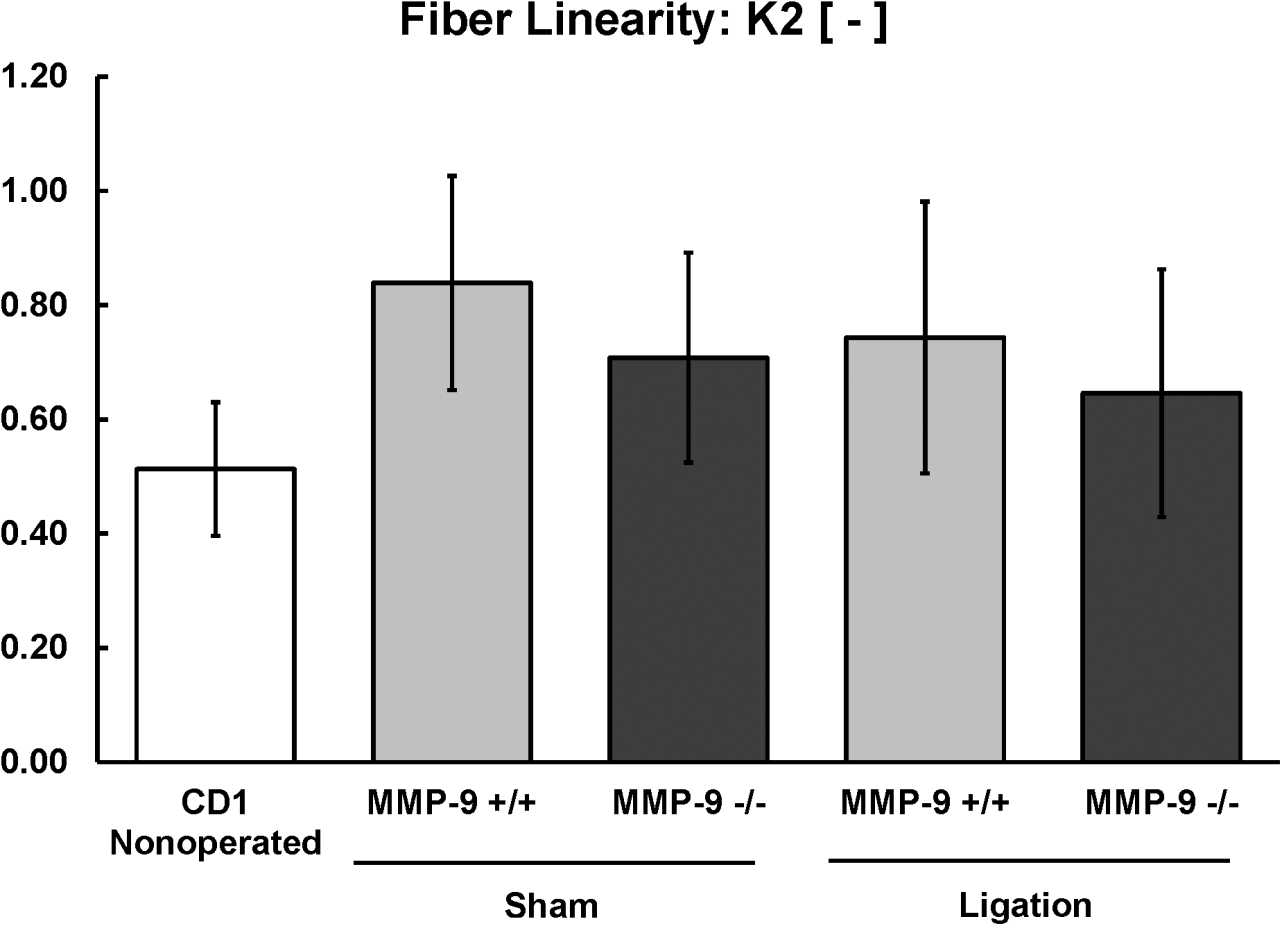
Biomechanical modeling of fiber linearity or organization (K2) of the post-thrombotic vein wall in MMP-9^+/+^ and MMP-9^-/-^ mice (N = 8–12 per group; p = 0.51).

**Fig 9:**
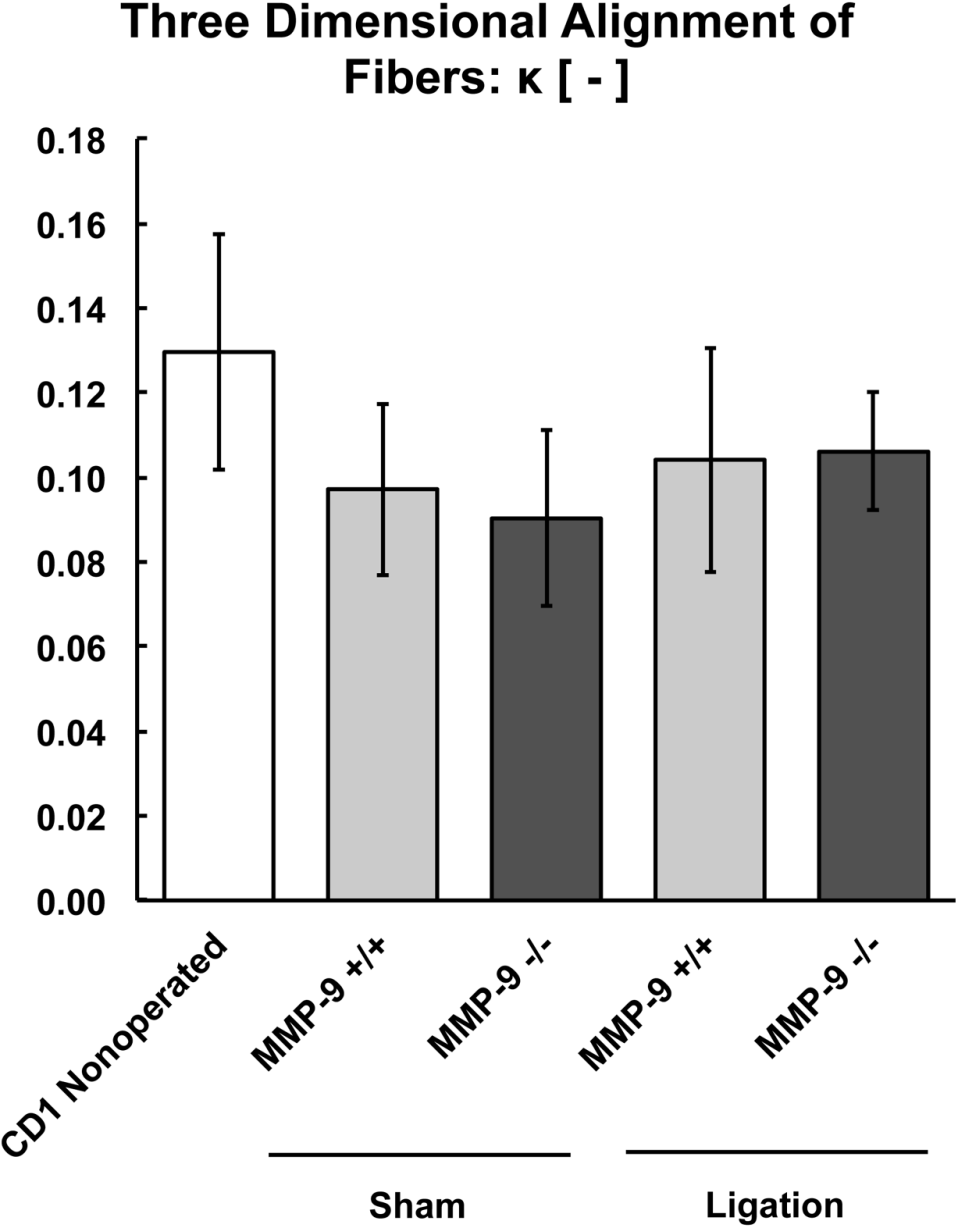
Biomechanical modeling of the three-dimensional alignment of fibers (κ) of the post-thrombotic vein wall in MMP-9^+/+^ and MMP-9^-/-^ mice (N = 8–12 per group; p = 0.72).

**Fig 10:**
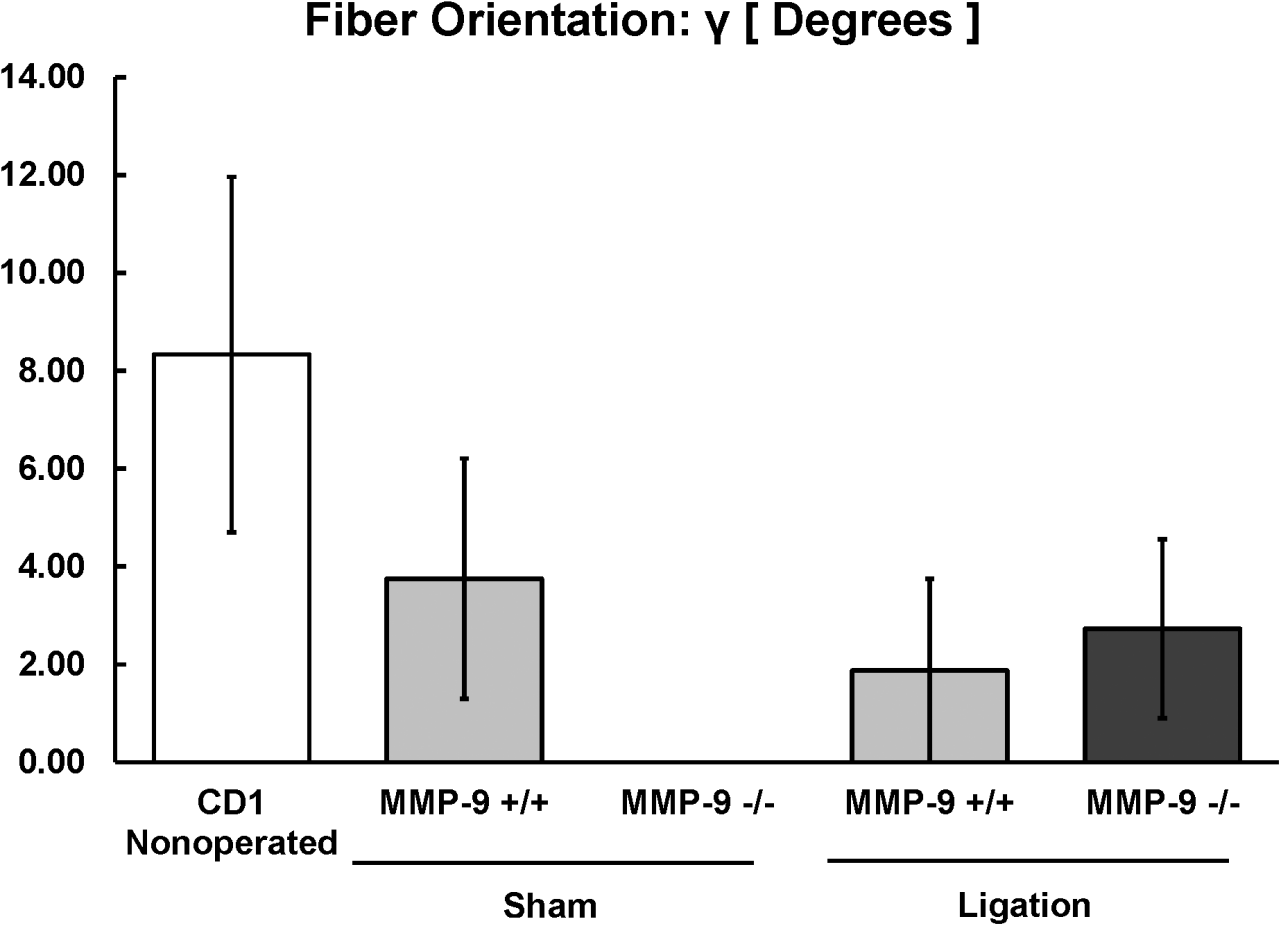
Biomechanical modeling of the contribution of collagen and elastin fiber orientation (γ) to the post-thrombotic vein wall in MMP-9^+/+^ and MMP-9^-/-^ mice (N = 8–12 per group; p = 0.84).

To define the effects of MMP-9 expression on vein wall compliance, we utilized novel biomechanical analysis techniques for the murine vein wall to define the effects of targeted deletion of MMP-9. The material and structural coefficients, represented in Figs 5–10, are a summary of statistically significant differences between MMP-9^+/+^ and MMP-9^-/-^ groups.

Overall vein wall compliance after sham surgery and thrombus resolution was represented as the inverse of the elastic modulus (MPa) in Fig 5. Sham surgery modestly increased the elastic modulus and this increase was dependent on MMP-9. Thrombus resolution significantly increased elastic modulus (MPa) in both MMP-9^+/+^ and MMP-9^-/-^ veins as compared to sham surgery, indicating that thrombus resolution decreased overall vein wall compliance of both MMP-9^+/+^ and MMP-9^-/-^ mice. Furthermore, MMP-9^-/-^ veins had significantly greater wall compliance as compared to MMP-9^+/+^ in both circumferential and axial directions after both sham surgery and thrombus resolution (Fig 5). Thus MMP-9 activity induced during thrombus resolution decreases vein wall compliance and increases stiffening in both axes of the vessel wall.

In both MMP-9^+/+^ and MMP-9^-/-^ mice, the C_10_ (extracellular matrix parameter) increased significantly in veins after thrombus resolution when compared with sham-operated veins (Fig 6). The C_10_ coefficient was significantly greater in sham-operated MMP-9^+/+^ mice than in sham-operated MMP-9^-/-^ mice (Fig 6), demonstrating that inflammation secondary to surgical manipulation increases stiffness of the non-fibrous extracellular matrix, and this increase is MMP-9 dependent. Similarly, lack of MMP-9 substantially attenuated (two fold) the more substantial increase in extracellular matrix stiffness induced by thrombus resolution (Fig 6).

In both MMP-9^+/+^ and MMP-9^-/-^ mice, the stiffness of collagen and elastin fibers (K_1_ parameter) increased significantly after thrombus resolution compared to sham-operated veins (Fig 7). The K_1_ coefficient was significantly greater in sham-operated MMP-9^+/+^ mice than in MMP-9^-/-^ mice (Fig 7), indicating that surgical manipulation of the IVC *per se* increased the stiffness of fibers in an MMP-9 dependent manner. During thrombus resolution, the K_1_ parameter increased over sham K_1_ values for both MMP-9^+/+^ and MMP-9^-/-^ mice with a significantly smaller increase in MMP-9^-/-^ mice (Fig 7). These findings suggest that MMP-9 increases stiffness of the collagen and elastin fibers during thrombus resolution.

There were no significant differences between the thrombi of MMP-9^+/+^ and MMP-9^-/-^ mice for fiber linearity (K_2_, Fig 8), three-dimensional alignment of fibers (κ, Fig 9), or orientation of collagen and elastin fibers (γ, Fig 10) to vein wall stiffness.

To summarize the biomechanical findings, both sham surgery and thrombus resolution caused stiffening of the vein wall with significantly greater stiffening noted after thrombus resolution. MMP-9^-/-^ mice had less overall stiffening and improved compliance of the vein wall, with the greatest effect of MMP-9 being on the extracellular matrix (C_10_) and a smaller but significant effect on collagen and elastin fibers (K_1_). MMP-9 affected stiffness induced by thrombus resolution in both the axial and circumferential directions of the vein wall.

### MMP-9 contributes to inflammatory cell recruitment and collagen metabolism in thrombus resolution

**Fig 11:**
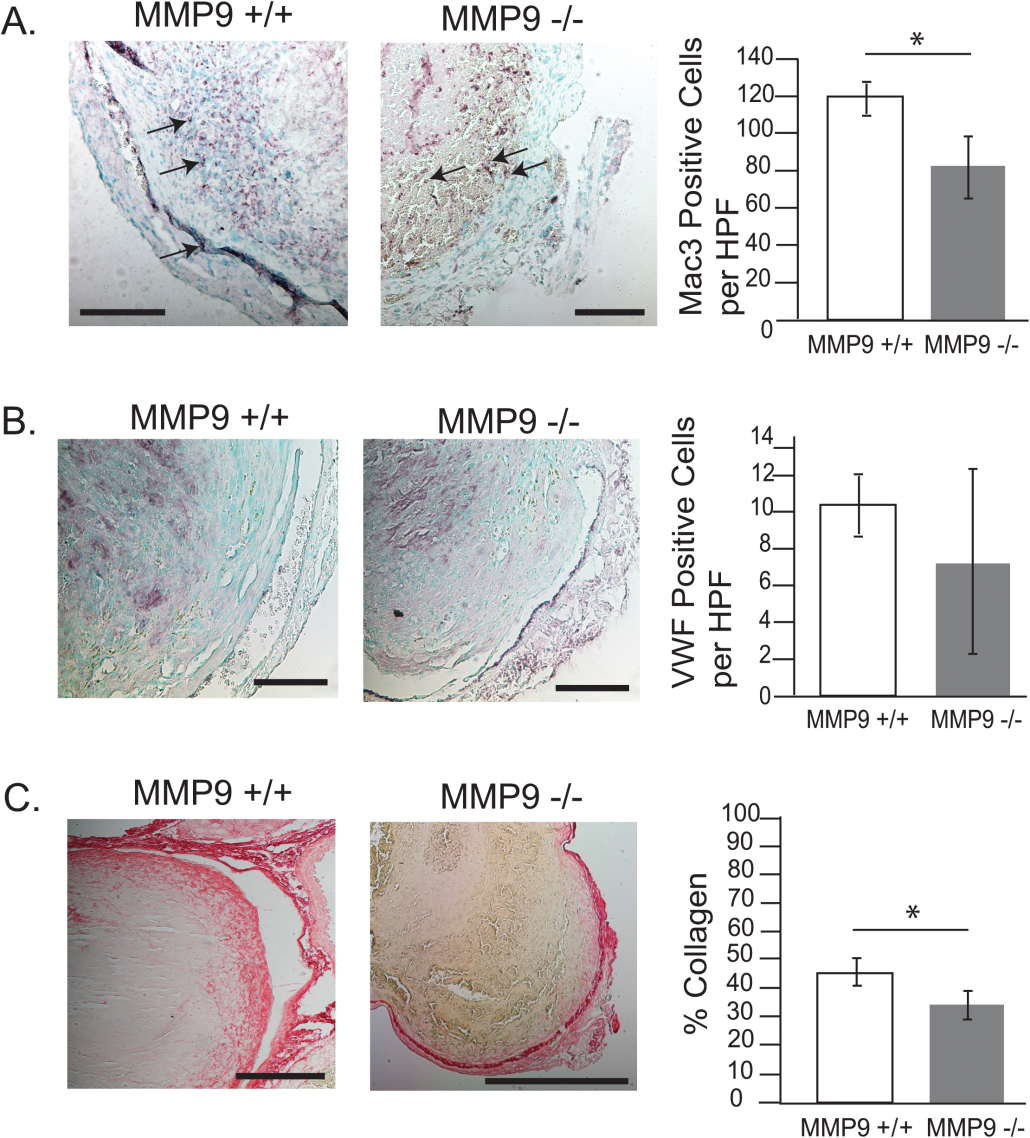
Immunohistochemical analysis of DVT in MMP-9^+/+^ and MMP-9^-/-^ mice. MMP-9 effects on thrombus cellularity and collagen. Macrophage counts (MMP-9^+/+^ 121±11, N = 4 versus MMP-9^-/-^ 85±18, N = 6; *p<0.05) (arrows indicate macrophages) (A), endothelial cell counts (MMP-9^+/+^ 10.7±2, N = 3 versus MMP-9^-/-^ 8.8±5, N = 5, p = 0.53) (B) and collagen content (MMP-9^+/+^ 45±5%, N = 4 versus MMP-9^-/-^ 34±4%, N = 6, *p<0.05) (C) in thrombi from MMP-9^+/+^ and MMP-9^-/-^ mice are shown on day 12 after IVC ligation. Six high powered fields (HPF) were evaluated per N and scale bar measures 100 uM.

A previous study has shown that MMP-9 alters collagen metabolism and expression of inflammatory fibrotic mediators [11]. To examine the cellular mechanisms underlying the biomechanical effects of MMP-9 on thrombus resolution, we compared the morphology of thrombi from MMP-9^+/+^ and MMP-9^-/-^ mice. There was a significant decrease in the number of macrophages in MMP-9^-/-^ as compared to MMP-9^+/+^ mice (Fig 11A). In contrast, von Willebrand factor (vWF) staining for endothelial cells showed no significant difference between MMP-9^-/-^ and MMP-9^+/+^ mice (Fig 11B). We quantified collagen as a percentage of total thrombus cross sectional area using picrosirius red staining. Comparison of thrombi on day 12 demonstrated that MMP-9^-/-^ mice had significantly less collagen than MMP-9^+/+^ mice (Fig 11C). MMP-9 mediation of macrophage infilitration and collagen metabolism during thrombus resolution is consistent with prior studies [11]. However, MMP-9 did not influence endothelial cell ingrowth after venous thrombosis.

### Role of bone marrow derived MMP-9 in thrombus resolution

**Fig 12:**
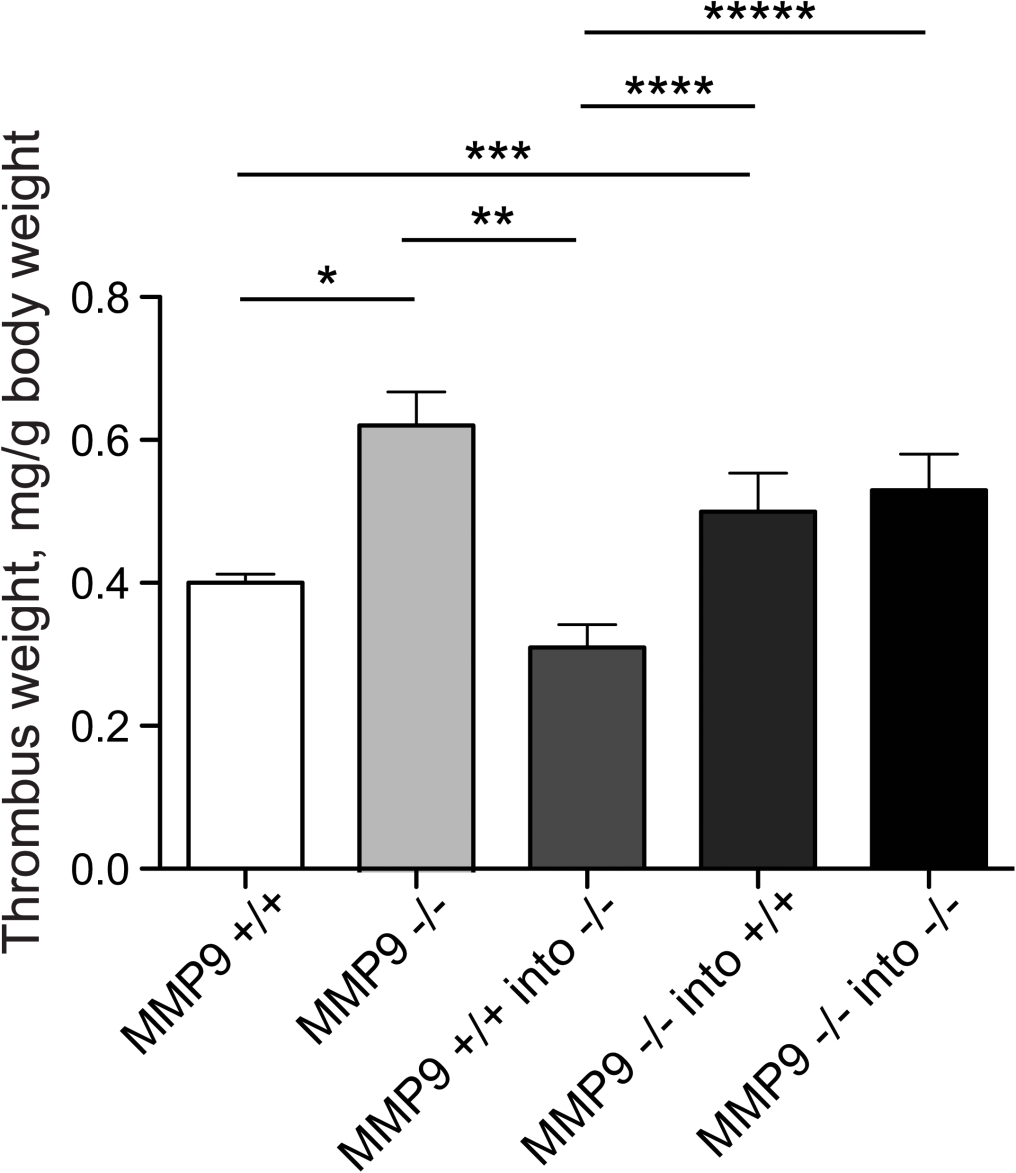
Role of bone marrow-derived MMP-9 in regulating thrombus resolution. Thrombus weights (g/kg) on day 12 after IVC ligation (DVT) are shown after bone marrow transplants between MMP-9^+/+^ and MMP-9^-/-^ mice (N = 6–10; *p<0.05. N = 10 per group; **, ***p<0.05.).

Since cells of bone marrow origin have been shown to play an essential role in thrombus resolution [29], we studied thrombus resolution in MMP-9^+/+^ and MMP-9^-/-^ mice, with each strain cross-transplanted with bone marrow from the other. MMP-9^+/+^ bone marrow cells accelerated thrombus resolution when transplanted into MMP-9^-/-^ mice (Fig 12). Conversely bone marrow cells lacking MMP-9 impaired thrombus resolution when transplanted into MMP-9^+/+^ mice (Fig 12). Amplification of Y chromosome loci by PCR was performed in all female recipient mice demonstrated positive and efficient engraftment of male donor marrow. Thrombus resolution was significantly impaired in all irradiated bone marrow recipients of MMP-9^-/-^ bone marrow cells as compared to recipients of MMP-9^+/+^ bone marrow cells (Fig 12). The effect of MMP-9 on thrombus resolution thus localizes to cells derived from bone marrow and the effect of MMP-9 on thrombus resolution can be reciprocally transferred by bone marrow transplantation. Additional controls were performed to account for the effects of irradiation and bone marrow transplantation itself on thrombus resolution. Thrombus resolution in irradiated MMP-9^-/-^ mice receiving MMP-9^-/-^ bone marrow cells was not different than non-irradiated, non-transplanted MMP-9^-/-^ mice (Fig 12), indicating that bone marrow transplantation *per se* did not influence thrombus resolution (N = 6–10, p = 0.4).

In summary, MMP-9 expression and activity were increased during thrombus resolution, and deletion of MMP-9 resulted in impaired thrombus resolution. Our biomechanical analysis demonstrate that MMP-9 mediates the decreased vein wall compliance induced by thrombus resolution and also has a role in collagen deposition and macrophage recruitment. The critical source of MMP-9 activity during thrombus resolution is bone marrow-derived cells, most likely macrophages.

## Discussion

This study demonstrates a novel and important role of MMP-9 in regulating biomechanical changes in the post-thrombotic vein wall. We identify the effects of MMP-9 on the structure and cellularity of the resolving thrombus, specifically its modulation of collagen and macrophage content. We also identify that bone marrow derived MMP-9 as a critical source in thrombus resolution. Using novel biomechanical analysis, we demonstrated that MMP-9 has opposing effects on two distinct and clinically relevant aspects of thrombus resolution, namely resolution of thrombus mass and stiffness of the vein wall. Prior biomechanical studies of vein wall stiffness after thrombus resolution were done in rats [8] [9], in which the roles that individual genes play on specific biomechanical parameters and vein wall components cannot be conclusively determined. Our findings are the first report of the specific biomechanical effects of a single gene during thrombus resolution. MMP-9 has a beneficial role in the resolution of thrombus mass but a detrimental role in the loss of compliance (stiffening) induced by thrombus resolution. Lack of MMP-9 reduced the overall stiffness induced by thrombus resolution by approximately 50% in both the axial and circumferential directions, and MMP-9 appears to have a greater effect on the stiffening of the extracellular matrix (C_10_) rather than collagen and elastin fibers (K_1_).

We find that the magnitude of the role of MMP-9 in thrombus resolution is similar to that reported for other molecular mediators of thrombus resolution *in vivo*. Specifically we note a 27% increase in thrombus size in mice lacking MMP-9, which is comparable in magnitude to changes noted during thrombus resolution in mice lacking TLR-9 (20% increase) [30], CXCR2 (40% increase) [22], MMP-2 (20% increase) [31], interferon-gamma (33% decrease) [32], uPA (25% increase) and PAI-1 (25% decrease) [33]. Our results differ from those of Deatrick et al. 2013 [11], who reported an 18% decrease in thrombus weights in mice deficient in MMP-9 in late thrombus resolution (day 21), whereas we report a 27% increase in thrombus size on day 12. Additionally, they report an increase in vein wall monocytes on day 8 but we report a decrease in total macrophages on day 12 in MMP-9^-/-^ mice. However, consistent with our results, they report a decrease in collagen content in MMP-9^-/-^ on day 8. Our direct measurements of vein wall biomechanics are also consistent with the decreased collagen content noted in both these studies in mice lacking MMP-9. These authors [11] attribute late thrombus resolution to the compensatory increase in MMP-2 activity in MMP-9^-/-^ mice. Differences in mouse background strain (FVB versus B6129 SvEv), time of analysis (day 12 versus 21), and method of calculating thrombus weights (thrombus weight normalized to body weight versus thrombus weight normalized to thrombus length) may contribute to these differences. We also used the more accurate method of comparing MMP-9^-/-^ mice to their littermate controls whereas they did not, raising the potential for genetic drift in the colony as another confounding cause of their findings. Given these divergent findings, it is interesting to note that our finding of a positive role for MMP-9 in thrombus resolution is also more consistent with the prior studies of uPA and gamma interferon in thrombus resolution. Thrombus resolution is enhanced in mice deficient in interferon-gamma and is associated with prolonged MMP-9 expressionwhereas mice lacking uPA have impaired thrombus resolution and marked decrease in MMP-9 [33] [34]. Thus our finding that MMP-9 mediates thrombus resolution provides a potential downstream mechanism for both the effects of interferon-gamma and uPA. Plasmin is a potential activator of proMMP-9 to its active form, and inhibition of plasmin during thrombus resolution results in decreased MMP-9 activity in the vein wall and increased thrombus size [9] [34]. Thus MMP-9 may also be a downstream mediator of the effects of plasmin on thrombus resolution. Patients with deep venous thrombosis have marked increases (>4 fold) in circulating MMP-9 [7], supporting its importance in clinical thrombus resolution.

Of the molecules proven to modulate thrombus resolution, a direct role for bone marrow-derived cells as the source has been shown only for uPA [34] and in this study for MMP-9. It is highly likely that the bone marrow-derived cells mediating the effects of uPA and MMP-9 (Fig 6) are macrophages, given the central role for macrophages in thrombus resolution [22] [35]. Inflammatory mediators and recruitment of macrophages and monocytes have a central role in thrombus resolution. The decreased macrophage infiltration in resolving thrombi from MMP-9^-/-^ animals is consistent with the key role of MMP-9 in macrophage migration *in vivo* in models of peritonitis, spinal cord injury and aortic aneurysms [36] [37] [18]. MMP-9 expression in macrophages is critical for migration through the type IV collagen present in the basement membrane of most tissues [37] [38] [18] [39]. In contrast, our findings of decreased macrophages in MMP-9^-/-^ mice are consistent with multiple other studies demonstrating increased macrophage infiltration into sites of inflammation [37] [38] [18] [39]. Macrophage MMP-9 expression affects vascular remodeling in other settings, including VEGF-induced angiogenesis in adult murine brains [40] and ischemia-induced angiogenesis in skeletal muscle [41]. It is also possible that MMP-9 activity during thrombus resolution is derived from neutrophils, as neutropenia is associated with impaired thrombus resolution and decreased intra-thrombus MMP-9 [42] [19].

Several specific factors and mediators contribute to the detrimental biomechanical changes (e.g. stiffening) of the vein wall induced by thrombus resolution. Inhibition of plasmin, a known proteolytic activator of pro-MMP-9, results in decreased vein wall stiffness and decreased MMP-9 activity [9]. Our finding of the pathological role of MMP-9 in mediating this vein wall stiffness provide a downstream mechanism for the effect of plasmin. Treatment with low molecular weight heparin decreases vein wall stiffness, but has no effect on resolution of thrombus mass [5]. In the same study, the MMP inhibitor doxycycline decreased MMP-9 expression but did not alter vein wall stiffness [5].

The decreased mechanical integrity of extracellular matrix and of collagen and elastin fibers in vein walls of MMP-9^-/-^ mice correlated with decreased amounts of collagen noted by immunohistochemical staining. However the magnitude of biomechanical changes exceeds the immunohistochemical differences in collagen content. This suggests that MMP-9 plays a greater role in remodeling of extracellular matrix and collagen fibers to induce vein wall stiffness than can be discerned by immunohistochemical analysis, and demonstrates that direct biomechanical analysis of mouse vein wall detects more subtle changes than immunochemistry. Standard quantitative techniques such as immunohistochemistry (Fig 11) or collagen quantitation [31] will not detect changes in the attachments of the collagen fibers to the basement membrane, crosslinking of non-fibrillar collagen or other biomechanically relevant structural alterations in the extracellular matrix proteins. We speculate that such changes are potentially responsible for the effects of MMP-9 on the extracellular matrix during thrombus resolution. Our finding that MMP-9 deletion decreases thrombus-induced stiffness of the vein wall is the first demonstration of a specific potential molecular target for the biomechanical damage induced by thrombus resolution.

Future studies will help further define the distinct beneficial and detrimental mechanisms of MMP-9 activity during thrombus resolution, namely the positive effect of MMP-9 on thrombus size and the detrimental effect on vein wall compliance. MMP-9 is a pro-inflammatory gene expressed in endothelial cells, smooth muscle cells and inflammatory cells in numerous settings of inflammation and tissue remodeling. While the beneficial effect of MMP-9 on resolution of thrombus mass may be inseparable from its role in promoting vein wall fibrosis and stiffening, it remains possible that these effects are due to distinct spatial and temporal effects of MMP-9 expression and activity. The finding that bone-marrow derived cells mediate the effect of MMP-9 on thrombus resolution is also relevant therapeutically as it suggests that progenitor cells or macrophages may be manipulated as a vehicle to increase MMP-9 expression within the thrombus.

## Conclusions

In this study, the precise role of MMP-9 in the loss of compliance during thrombus resolution was delineated by biomechanical analysis of vein walls in mice lacking MMP-9. This novel experimental approach demonstrates distinct and opposite effects of MMP-9 on clinically relevant parameters of thrombus resolution, and could be similarly applied to the other proteases and inflammatory mediators expressed during thrombus resolution *in vivo* [43] [44]. Our findings regarding MMP-9 as a mediator of thrombus resolution provide a unifying molecular mechanism for the changes in thrombus resolution and vein wall stiffening noted in a variety of prior studies, including those of gamma-interferon [32], uPA [33] [34], neutropenia [42] [19] and plasmin inhibition [9]. Defining the effects of individual mediators on both thrombus size and vein wall biomechanics will aid in the development of specific molecular therapy for deep venous thrombosis. Such therapy may augment thrombus resolution, preserve vein wall compliance and potentially decrease secondary complications such as post-thrombotic syndrome.

## Supporting Information

S1 ARRIVE Guideline Checklist (PDF)

S1 Data (XLSX)
